# Research activities and critical appraisal skills among Saudi orthopedic residents

**DOI:** 10.1186/s12909-021-02772-y

**Published:** 2021-06-02

**Authors:** Omar A. Al-Mohrej, Nouf F. Alsadoun, Fawaz N. Alshaalan, Rayan I. Alturki, Anwar M. Al-Rabiah, Nezar B. Hamdi, Imran Ilyas, Thamer S. AlHussainan

**Affiliations:** Department of Orthopedics, King Faisal Specialist Hospital and Research Center, Riyadh, Saudi Arabia

## Abstract

**Background:**

Medical research is a central part of any residency training. In view of the new Saudi orthopedic committee promotion regulation that mandates each resident to participate in a research project, the challenges that stand in the way of completion of substantial research within surgical residency must be investigated. The aim of this study was to assess the practice, attitudes, perception, and limitations associated with research among residents in the Saudi orthopedic program in the central region.

**Methods:**

A cross-sectional study was conducted between June and July 2020 using an online-based survey. The total number of study participants was 128 orthopedic residents out of the 191 residents enrolled in the central region program. Data were analyzed, and descriptive statistics in the form of frequency and percentage were determined, analytical tests were performed with P < 0.05 being statistically significant.

**Results:**

Most residents (95 %) participated in a research project during residency. Most projects (53.10 %) were case reports followed by retrospective studies (48.40 %). The majority (79.70 %) did not attend a research methods course during residency. Experience in research differed significantly (P < 0.05) by age, residency year, and center. The mean involvement score was significantly higher among males at 3 (± 1) than among females at 2 (± 0) (*P* < 0.001). Only 40.60 % have access to orthopedic journals, and the same percentage (40.60 %) knew how to Critique original articles. There was a statistically significant difference in the accessibility score according to the training center. Lack of faculty support and mentorship were the main barriers to medical research at 62.50 and 39.10 %, respectively. A total of 68.80 % reported that funding was not available through their institutes.

**Conclusions:**

In Saudi Arabia, the level of meaningful clinical research and publications by orthopedic residents is still low. The results of this study should be taken into consideration before the implementation of the new promotion criteria in the centers under the umbrella of Saudi orthopedic committee.

**Supplementary Information:**

The online version contains supplementary material available at 10.1186/s12909-021-02772-y.

## Introduction

The discipline of orthopedic surgery has continued to progress through evidence-based research, which has enabled physician instructors to continually educate residents on improving musculoskeletal patient care. Such research is perceived by many people as a central part of any residency program [1]. Nonetheless, it is a more contentious prerequisite for orthopedic residency programs within the field of scholarly activity [2].

The Saudi orthopedic residency program has assimilated a component of research training into their study modules. Initially a mandatory three-month research course was a part of the training curriculum that had to be taken during the first 2 years of residency. This was later replaced by Monthly journal club meetings and lectures where residents, both juniors and seniors are taught how to dissect and critique literature [3].

However, the various challenges concerning the completion of substantial research within surgical residency programs have been illustrated alongside drawbacks in time, research funding, and professional mentors [4]. Additional challenges to an extensive research experience in residency involve limited organizational infrastructure, deficiency priority, financial limitations on residency budgets, and inadequate guidance for Saudis to develop into clinical scientists [5].

Recently, in Saudi Arabia, the scientific committee of the orthopedic residency initiative in the Saudi Commission for Health Specialties recommended that research be mandatory for the natives to be promoted to the following level. Nonetheless, not much is known about their perception, practice, attitude, and limitations towards these natives’ research. As far as we know, no research has evaluated the attributes of the limitations encountered by Saudi orthopedic residents while conducting research.

Therefore, in this study, our objective was to assess the practice, attitudes, perceptions, and limitations associated with research among residents in the Saudi orthopedic program and establish factors that affect them, such as educational background, demographic components, and research involvement.

## Materials and methods

### Study design and setting

This cross-sectional study was conducted between June and July 2020 using an online-based survey, among Orthopedic training residents enrolled in the Saudi orthopedic training program in the central region of Saudi Arabia. The total number of orthopedic surgeons in Saudi Arabia was 2,179 (Both Board certified and still under training). Out of this number, only 189 Board certified orthopedic surgeons are citizens of the capital city of Saudi Arabia, Riyadh [6]. However, the number of orthopedic residents currently under training, in Riyadh, was 191 which is our target population [7].

### Recruited centers:

Online based Survey was sent to all Residents from the 10 training centers available in Riyadh were recruited in this study. These centers included: King Abdulaziz Medical City (KAMC), Security Forces Hospital (SFH), King Faisal Specialist Hospital and Research Center (KFSHRC), King Saud Medical City (KSMC), Habib Medical Group (HMG), Iman General Hospital (IGH), King Fahad Medical City (KFMC), King Fahad Hospital Medina (KFH-M), King Khalid University Hospital (KKUH), Prince Sultan Military Medial City (PSMMC).

### Data collection

The investigators in this study had previous experience in the field and since no similar studies existed before in Saudi Arabia, a questionnaire was formulated specifically for this study based on previous study [8]. The questionnaire used in the study had five divisions. These divisions were used to draft the questionnaire (Additional file 1).

In the first division, information about the demographic and educational status of the participants was covered. Some of these data included gender, age, and training level. In the second division, the investigators examined the participants’ experiences with medical research. It also included information about how the resident learned of the researchers, their participation, and the approximated time spent per month while conducting these studies. During the third part, the investigator assessed the participant’s involvement in research publication and presentation. The fourth division included checking of the citizen’s accessibility to orthopedic journals. It also included information about these citizens’ ability to understand and criticize pieces of evidence recorded in articles. The final part was divided into three subsections that addressed various types of information including the value of medical research, complexity, perceptions of medical research, and barriers to medical research such as limited time, lack of statistical knowledge, unavailability of mentorship, and lack of funding.

The above subsections were examined using the Likert scale ranging from 1 to 5. Each number presented a certain degree, as indicated (1 = strongly disagree, 2 = disagree, 3 = neutral, 4 = agree, 5 = strongly agree). Based on the above measures, we categorized the participants’ responses to all questions regarding barriers, perceptions, and attitudes into positive and negative answers. Any statements that did not favor medical research corresponds to strongly disagreeing or disagreeing. However, those with positive answers responded to strongly agreeing, agreeing, or neutral [8, 9].

The questionnaire was pilot tested to determine its clarity and reliability in examining the appropriateness of the questions. The questionnaires were administered to 15 applicants of orthopedic surgery in Jeddah area. The participants’ feedback was mainly based on the structure of the questionnaire. Also, some answers were based on the ambiguity of the original question. The comments developed after the questionnaire were considered; thus, the questionnaire was modified to create the final draft. The final draft was dispersed in the student sample. However, the results were not part of the final data analysis of the study.

To assess face and content validity, a panel of orthopedic experts in medical education and research evaluated the survey items, and the items were modified according to the experts’ ratings and suggestions. The content validity index (CVI) was used to assess items validity and experts were asked to rate each item based on the relevance. The CVI ranged 0.80 from 1.00 and the total CVI was 0.91, indicating content validity was acceptable [10]. Cronbach’s alpha coefficient was used to approximate internal consistency [11]. From the questionnaire items, a 0.7 internal consistency was achieved, which was good. Difficulty and discrimination items were used in item analysis. An item’s difficulty is the percentage of the individual who responds to the assessment item appropriately, with a higher score demonstrating a lower difficulty. These items demonstrated a range of 0.02–0.86, which indicates low to medium difficulty. Other items verified a higher discrimination index (0.1–0.5), indicating that the item is of good quality.

Before the study was conducted, the residents were informed their participation would be requested, but it was completely voluntary with no direct benefits. They were also informed that they could skip any questions they wished not answer and were allowed to opt out at any point in the study. The participants who had initially filled a questionnaire from previous research were prohibited from participating again. Later, the participants who wished to participate were administered a 15-minute questionnaire by the coinvestigators and left for their privacy and anonymity.

### Selection of participants

The sample size was not derived from statistical grounds instead of convenience, as the study’s main goal was descriptive. The aim of the study was explained through Google forms, and links were sent via email. Before participation, all participants were asked to complete an informed consent, which indicated their rights during the study and the research purpose. The number of eligible residents was 191 [12]. Of those, 128 has participated with rate of the responses recorded from the survey was 67.0 %. Those who did not reply to the online survey or clicked on the link but did not complete the survey were considered non-responders.

### Statistical analyses

After the data were collected, they were fed into Excel and later moved to the statistical package for social sciences (SPSS) software version 24. The data on categorical variables were determined using numbers and percentages. Standard deviation (SD) was used for continuous variables. The constant variables with more than two categories and an independent t-test were analyzed using a one-way analysis of variance (ANOVA). A bivariate analysis of the questionnaire section, that is, barriers, attitudes, perceptions, research involvement, and demographic characteristics, was examined. P < 0.05 was considered statistically significant.

## Result

The total number of participants was 128 orthopedic residents with a mean (± SD) age of 27.56 (± 1.96), most were males at 122 (95.30 %), and the highest number was in R2 at 36 (28.10 %). The highest proportions of the participants were from King Saud Medical City (KSMC) at 18.80 %, followed by King Abdulaziz Medical City (KAMC) and King Faisal Specialist Hospital (KFSHRC) at 15.60 % each (Table 1).

**Table 1:** Characteristics of Study Sample

Categories	Variables	N	%
Gender	Male	122	95.30 %
	Female	6	4.70 %
Age	Mean ± SD	27.56 ± 1.967	
Training level	R-1	22	17.20 %
	R-2	36	28.10 %
	R-3	30	23.40 %
	R-4	26	20.30 %
	R-5	14	10.90 %
Training center	KAMC	20	15.60 %
	SFH	12	9.40 %
	KFSHRC	20	15.60 %
	KSMC	24	18.80 %
	HMG	4	3.10 %
	IGH	6	4.70 %
	KFMC	10	7.80 %
	KFH-M	10	7.80 %
	KKUH	14	10.90 %
	PSMMC	8	6.30 %

More than half (51.60 %) of the respondents participated in research in medical school, and the experience was not significantly higher among males compared to females at 4 (± 2) vs. 3 (± 1), respectively, with a P value of 0.80. Conversely, experience in research differed significantly (P < 0.05) by age, residency year, and center, as shown in Table 2. The mean of the total hours per month the participating residents dedicated for research was 5.42 (± 9.67), and the number of projects they completed or were involved in was 1.05 (± 1.84).

**Table 2:** Factors affecting Research experience

Categories	Variables	Number	Participant’s Experience Score	
			Mean ± SD	*P*-Value
Gender	Male	122	4 ± 2	0.802
	Female	6	3 ± 1	
Age	-		-	**0.007**
Training level	R-1	22	5 ± 2	**0.004**
	R-2	36	4 ± 2	
	R-3	30	3 ± 2	
	R-4	26	3 ± 1	
	R-5	14	3 ± 2	
Training center	KAMC	20	5 ± 2	**< 0.001**
	SFH	12	5 ± 2	
	KFSHRC	20	4 ± 1	
	KSMC	24	3 ± 2	
	HBG	4	3 ± 1	
	IGH	6	5 ± 2	
	KFMC	10	4 ± 1	
	KFH-M	10	2 ± 2	
	KKUH	14	2 ± 2	
	PSMMC	8	2 ± 1	

Almost 56 % of the respondents had written their proposal application, while the majority (79.70 %) did not attend a research methods course during residency, and 68.80 % reported that funding is not available either through their university program or resident research projects. The highest residency research project participants either planned or already involved in was a case report at 53.10 %, followed by a retrospective study at 48.40 %, while 4.70 % had never participated in research projects. The mean involvement score was significantly higher among males at 3 (± 1) compared to 2 (± 0) for females with *P* < 0.001. The involvement scores also differed significantly by training level and center, being the highest in R1, R2, KAMC, and SFH at 4 (± 2), 4 (± 1), 5 (± 5), and 5 (± 1), respectively. The data are shown in Table 3. The mean involvement in peer-reviewed publications in the current study was 1.14 (± 2.17), and the mean number of peer-reviewed publications in which participants were the first author was 0.45 (± 1.68).

**Table 3:** Factors affecting research involvement

Categories	Variables	Number	Participant's Involvement Score	
			Mean ±SD	*P*-Value
Gender	Male	122	3±1	< .001
	Female	6	2±0	
Age	-		-	0.001
Training level	R-1	22	4±2	< .001
	R-2	36	4±1	
	R-3	30	3±1	
	R-4	26	3±1	
	R-5	14	3±1	
Training center	KAMC	20	5±2	< .001
	SFH	12	5±1	
	KFSHRC	20	3±1	
	KSMC	24	3±1	
	HBG	4	4±1	
	IGH	6	4±1	
	KFMC	10	2±1	
	KFH-M	10	3±1	
	KKUH	14	3±2	
	PSMMC	8	3±1	

Approximately 57.80 % of the residents had never presented a research project, but for those who presented, the highest percentages were in international and regional conferences at 18.80 % for each. In their scientific talk/research, participants reported that they were mostly dependent on ortho-bullets, up-to-date, and Medscape at 32.80 %, and only 39.10 % put references in each slide in the presentation. More than three quarters (76.60 %) reported that they read updated articles, only 40.60 % had access to orthopedic journals, and the same percentage (40.60 %) knew how to criticize original articles. There was a statistically significant difference in the accessibility score by the training center, where the high accessibility was in the KFSHRC, KSMC, and Habib Medical Group (HMG), as shown in Table 4.

**Table 4:** Factors affecting Research Accessibility

Categories	Variables	Number	Accessibility Score	
			Mean ± SD	*P*-Value
Gender	Male	122	2 ± 1	0.069
	Female	6	3 ± 1	
Age	-		-	0.158
Training level	R-1	22	2 ± 2	0.079
	R-2	36	2 ± 1	
	R-3	30	2 ± 1	
	R-4	26	3 ± 1	
	R-5	14	3 ± 1	
Training center	KAMC	20	2 ± 1	0.01
	SFH	12	2 ± 1	
	KFSHRC	20	3 ± 1	
	KSMC	24	3 ± 1	
	HBG	4	3 ± 1	
	IGH	6	1 ± 1	
	KFMC	10	2 ± 1	
	KFH-M	10	2 ± 1	
	KKUH	14	2 ± 1	
	PSMMC	8	2 ± 1	

The overall barrier score was 34 (± 7) out of 50, which indicates that residents were between “neutral” and “agree” on the assessed barriers. Participants were mostly “strongly agree” in which constrains due to residency duty and lack of faculty support and mentorship are barriers to medical research at 62.50 and 39.10 %, respectively. Ethical approval and lack of personal interest were not major barriers to research, as per the current study results. More than half of the respondents were between either “agree” (32.80 %) or “strongly agree” (26.60 %), where lack of statistical knowledge is a barrier to medical research. Equipment availability and lack of relevant research questions represented barriers according to almost 40 and 44 % of orthopedic residents in the current study, respectively. The total means of the barrier scores were not statistically significant in terms of gender, training level, training center, and age. Data are shown in Table 5.

**Table 5:** Research Barriers Among Residents

		Number	Barriers Score	
			Mean ± SD	*P*-Value
Gender	Male	122	34 ± 7	0.242
	Female	6	35 ± 2	
Age	-		-	0.274
Training level	R-1	22	34 ± 5	0.137
	R-2	36	36 ± 7	
	R-3	30	32 ± 6	
	R-4	26	34 ± 6	
	R-5	14	35 ± 8	
Training center	KAMC	20	33 ± 6	0.068
	SFH	12	34 ± 5	
	KFSHRC	20	33 ± 7	
	KSMC	24	33 ± 8	
	HBG	4	28 ± 3	
	IGH	6	42 ± 3	
	KFMC	10	34 ± 5	
	KFH-M	10	34 ± 9	
	KKUH	14	36 ± 5	
	PSMMC	8	35 ± 6	

For attitude and perception, the overall mean score was 45 (± 9). Male gender showed a statistically significant positive attitude towards medical research at 45 (± 9), which is in the area between “agree” and “strongly agree,” compared to 39 (± 3) for females, which is in the area between “neutral” and “agree” with *P* < 0.001. The mean total score of attitude and perception differed significantly (*P* = 0.002) according to the residency level and ranged from 41 (± 9) to 48 (± 7). In contrast, there was no significant difference between the participating centers with regard to the total mean score of attitude and perception, as shown in Table 6.

**Table 6:** Factors Affecting the Attitude and Perception toward medical Research

		Number	Attitude and perception	
			Mean ± SD	*P*-Value
Gender	Male	122	45 ± 9	0.001
	Female	6	39 ± 3	
Age	-		-	0.865
Training level	R-1	22	42 ± 10	0.002
	R-2	36	48 ± 7	
	R-3	30	41 ± 9	
	R-4	26	47 ± 7	
	R-5	14	45 ± 8	
Training center	KAMC	20	47 ± 8	0.135
	SFH	12	47 ± 6	
	KFSHRC	20	45 ± 6	
	KSMC	24	43 ± 12	
	HBG	4	47 ± 5	
	IGH	6	51 ± 7	
	KFMC	10	49 ± 4	
	KFH-M	10	39 ± 12	
	KKUH	14	43 ± 6	
	PSMMC	8	42 ± 5	

## Discussion

The importance of research exposure and education during orthopedic residency has been discussed over the past few decades [13, 14]. The findings of this study, however, show that the level of engagement in research activities by orthopedic residents is still poor. Research in healthcare is known to have a high significance in addressing several gaps, which include the elimination of obstacles towards provision of quality care, improvement of evidence-based practice, increased patient satisfaction, and positive health outcomes. [15].

In this study, half of orthopedic residents reported having participated in research activities at school. These findings showed that only half of orthopedic residents considered the research activities to be useful to their studies and work. On the contrary, nearly half of orthopedic residents did not value research activities while at their schools.

The findings further reveal that during their residency, 56 % of orthopedic residents did not write their proposal application. In addition, 79.70 % of the applicants failed to attend any research activities. The results indicated that the level of engagement in research activities among orthopedic residents was low. Several factors could contribute to participation in research activities among orthopedic residents. Inadequate finances are among the leading factors for reduced engagement in research activities among orthopedic residents [16]. In this study, the results reveal that 68.80 % of participants did not have funds for their research activities. Another contributing factor is the lack of support from faculty. In this review, 62.50 % of participants reported that they did not have faculty support and mentorship, which limited their participation in research activities.

Jain [17] notes that the developed world has been guiding research activities in orthopedic practices leading to a low disease profile compared to other nations. Through research activities involving orthopedics, Western countries have managed to eliminate some diseases [17]. In developing areas, 2/3 of the population still encounters a natural history of diseases due to poor research activities.

Al-Haiden et al. evaluated the training program for orthopedic residency in Saudi Arabia in comparison to a Canadian program [18]. The authors reveal that in the two countries, specialty information originates from textbooks. However, unlike in Saudi Arabia, orthopedics in Canada tend to be highly scholarly. As per the study findings, while 46.7 % of orthopedics in Canada read scholarly journals or articles, only 10.5 % of orthopedics in Saudi Arabia read them [18]. These findings are closely related to the current study, where the source of information is textbooks. Makhdom et al. reported that engagement in clinical orthopedic research was also low in Saudi Arabia [19]. A review involving 91 orthopedic surgeons and 251 articles showed that the evidence collected within 20 years was low compared to the studies conducted in North America [19]. A leading factor for the sparse evidence in orthopedic research conducted in Saudi Arabia was inadequate funding to support research assistants. Therefore, most orthopedic surgeons focused on retrospective case studies that did not involve many people. The quality of such research is usually poor [19]. A similar trend in poor orthopedic research was common among orthopedic residents. Lack of adequate support, including poor mentorship, low finances, and lack of personnel, discourages orthopedic residents from active engagement in research activities [4, 5].

The outcome of this study also showed that participation in research publications was low among orthopedic residents in Saudi Arabia. A related study conducted by Campbell et al. showed that the number of orthopedic residents who participated in research and journal publications was small [20]. Inadequate skills in clinical research and lack of interest are among the factors that make it difficult for orthopedic residents to participate in research activities and publications.

Training programs could also influence the participation of orthopedic residents in research activities. Compared to other training centers outside Riyadh, residents in Riyadh have reduced reliance on textbooks as a source of information during their training [16]. Riyadh residents have increased the use of peer-reviewed scholarly articles to support their training and knowledge as compared to residents in other training centers [16]. Despite that, Orthopedic residents in Riyadh still don’t participate in research publication in a meaningful way compared to other training programs in the country. Therefore, differences in the programs in Saudi Arabia are there, but not to that extent.

Lack of interest among orthopedic residents is one of the factors that make it challenging to include research activities in training programs [5]. The interest in research activities emanates from factors such as older age, participation in manuscript authorship, and previous research experience. Despite Most orthopedic surgeons globally are considered clinician-scientists, this doesn’t seem like the case in Saudi Arabia [5]. Therefore, motivating them could increase their interest in research activities. However, participation in research activities depends mainly on the priority of orthopedic residents.

Inadequate infrastructure and finances within the training centers were also another challenge for the inclusion of research activities in orthopedic residency training programs [21]. A supportive environment for research depends on the availability of infrastructure and adequate financial resources. Institutions in Saudi Arabia, particularly the Riyadh Training Center, need to invest in facilities, for instance, establishing advanced biomechanics laboratories, which will encourage orthopedic residents to participate in research activities [22]. Recruiting qualified personnel in clinical research would also increase participation in research activities.

Two limitations could have negatively influenced the outcome of this study. First, the selected sample size was small compared to the target study population. The number of orthopedic residents in Saudi Arabia is increasing; hence, a small sample size could fail to represent all of them. With a small sample, chances of variability and bias in results could be high, thereby making the outcome unreliable. Second, the study involved only one survey instrument to collect the data. The validity and reliability of the instrument used have not been well established. With the questionnaire used, the chances of researcher bias are high, which could affect the accuracy and reliability of the results.

## Conclusions

In Saudi Arabia, the level of participation in clinical research and publications by orthopedic residents is still low. Only half of orthopedic residents engage in research activities and publications. The leading factors for poor participation in research activities include lack of interest among orthopedic residents, inadequate funding, inadequate research infrastructure at the training centers, and poor mentorship or support from qualified personnel. Thus, the results of this study should be taken into consideration before the implementation of the new promotion criteria in the centers under the umbrella of SCFHS.

## Supplementary Information

**Additional file 1.** Study Questionnaire.

## Data Availability

The datasets used and/or analyzed during the current study are available from the corresponding author on request.
