# Supplementary material for: Research activities and critical appraisal skills among Saudi orthopedic residents
Source: BMC Med Educ. 2021 Jun 2;21:311. doi: 10.1186/s12909-021-02772-y (PMC8173921; doi:10.1186/s12909-021-02772-y)
Supplement: Supplementary file 1 — Additional file 1. Study Questionnaire. [file 12909_2021_2772_MOESM1_ESM.doc]

Research activities and critical appraisal skills among Saudi orthopedic residents

- - Required

1. Age *

in years, number


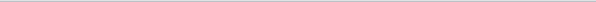


1. Gender *

***Mark only one oval.***


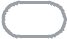
 Female


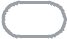
 Male

1. Training level *

***Mark only one oval.***


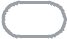
 R-1


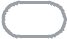
 R-2


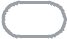
 R-3


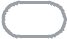
 R-4


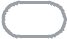
 R-5

1. Please, write your training center name. *


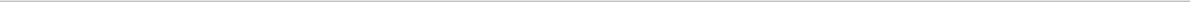

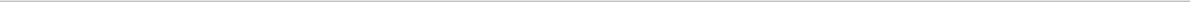

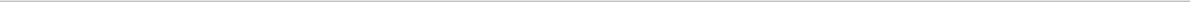

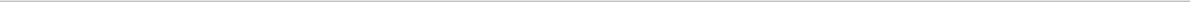

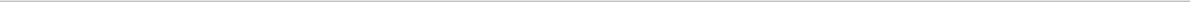


1. What previous experience do you have with research? *

***Mark only one oval.***


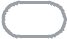
 Masters degree


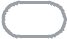
 Phd


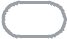
 Worked in research prior to residency


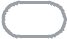
 Participated in research in medical school


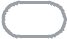
 Participated in research during undergraduate education


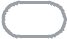
 Completed an undergraduate thesis


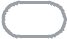
 None

1. Have you ever written your own Proposal application? *

***Mark only one oval.***


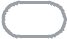
 Yes


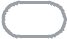
 No

1. Have you attended a research methods course during residency? *

***Mark only one oval.***


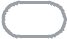
 Yes


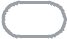
 No

1. On average, how many hours per month do you estimate you dedicate towards

research? *

in hours, number


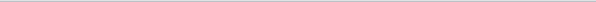


1. How many resident research projects have you completed or are currently involved in (total)? *


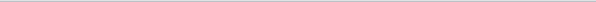


1. Is funding available through your university program or university for resident research projects? *

***Mark only one oval.***


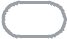
 Yes


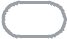
 No

1. Is there protected research time as part of your residency? *

***Mark only one oval.***


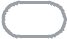
 Yes


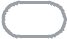
 No

1. Does your program have or participate in a resident research day? *

***Mark only one oval.***


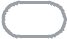
 Yes


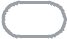
 No

1. Does your research supervisor publish at least once per year? *

***Mark only one oval.***


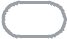
 Yes


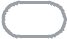
 No

1. Have you published in a peer-reviewed journal during residency? *

***Mark only one oval.***


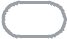
 Yes


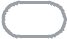
 No

1. Have you published in a peer-reviewed journal prior to residency? *

***Mark only one oval.***


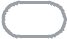
 Yes


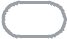
 No

1. How many peer reviewed publications have you been involved in? *
2. How many peer-reviewed publications were you first author on? *
3. My planned resident research project/current resident research project includes involvement in: *


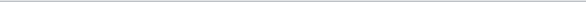

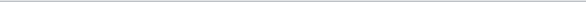


***Check all that apply.***


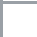

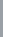
 Case report


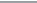


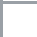

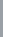
 Prospective study


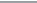


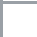

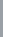
 Retrospective study


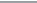


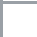

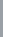
 Basic science project


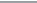


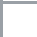

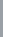
 Survey study


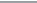


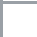

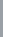
 Use of a database (which was not compiled by me)


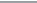


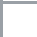

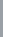
 Literature review


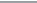


Other:
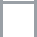


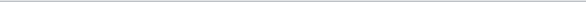

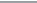


1. Do you read updated articles as part of knowledge resources ? *

***Mark only one oval.***


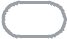
 Yes


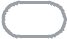
 No

1. Do you have an access to orthopedic journals? *

***Mark only one oval.***


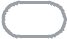
 Yes


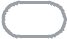
 No

1. Do you know/read the respected journals in orthopedic surgery? *

***Mark only one oval.***


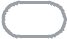
 Yes


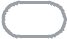
 No

1. Do you know how to criticize the evidence presented in original articles? *

***Mark only one oval.***


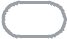
 Yes


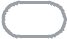
 No

1. Which of the following are barriers to completion of research during your residency? *

***Mark only one oval per row.***

***
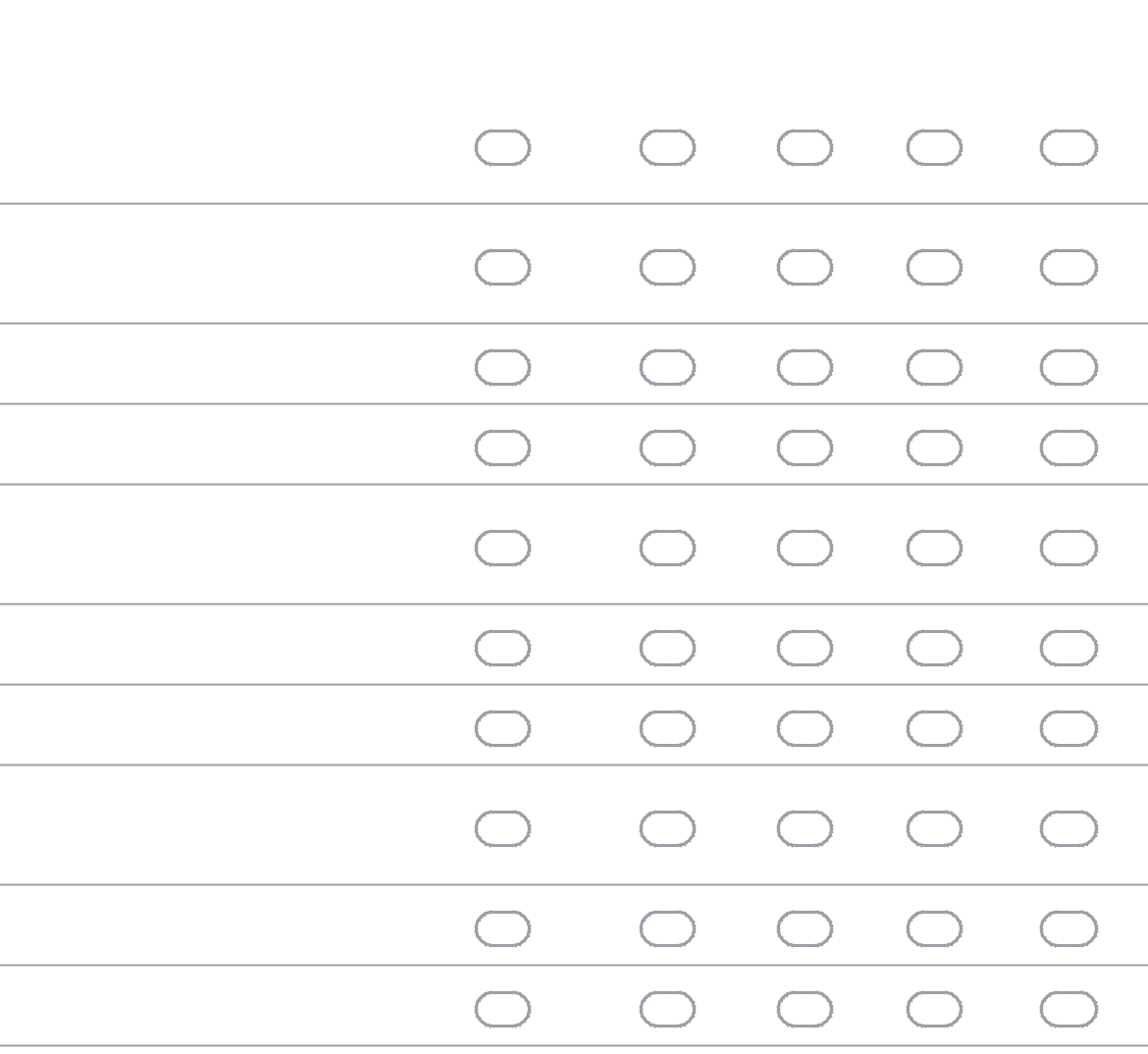
***

| Strongly | Disagree Neutral | Agree | Strongly |
| --- | --- | --- | --- |
| disagree |  |  | agree |
|  |  |  |  |
|  |  |  |  |

**T**i**me constra**i**nts due to**

**res**i**dency dut**i**es**

**T**i**me constra**i**nts due to**

**persona**l **comm**i**tments**

**Lack of persona**l i**nterest**

**Lack of fund**i**ng**

**Lack of facu**l**ty**

**support/mentorsh**i**p**

**Eth**i**cs approva**l

**Lack of stat**i**st**i**ca**l **know**l**edge**

**Lack of re**l**evant research**

**quest**i**on**

I**nab**ili**ty to recru**i**t pat**i**ents**

**Equ**i**pment ava**il**ab**ili**ty**

1. I have presented research at a: *

***Check all that apply.***


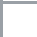

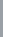
 International conference


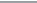


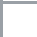

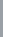
 National conference


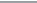


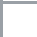

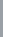
 Regional conference


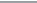


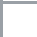

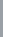
 Resident research day


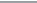


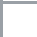

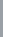
 I have not presented research at any of these places


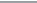


1. When you present a scientific talk/research, in which of the following do you depend most frequently *

***Mark only one oval.***


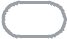
 Well known books, such as miller, handbook of fractures, Netter .. etc
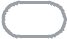
 Orthobullets, up-to-date, medscape .. etc


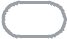
 Review articles and books such as AAOS comprehensive Orthopedic review, EFORT review .. etc


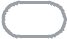
 Up-to-date scientific original/meta-analysis/systematic-review articles in respected journals such as NJEM, JBJS, BJR .. etc


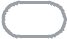
 Other:


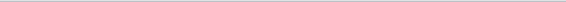


1. Do you put your references in each slide in the presentation ? *

***Mark only one oval.***


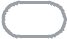
 Yes


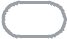
 No

10/18/2020 Survey on Research Activities and Critical appraisal in Orthopedic Programs

1. Please rank your agreement with each of the following statements: *

***Mark only one oval per row.***

***
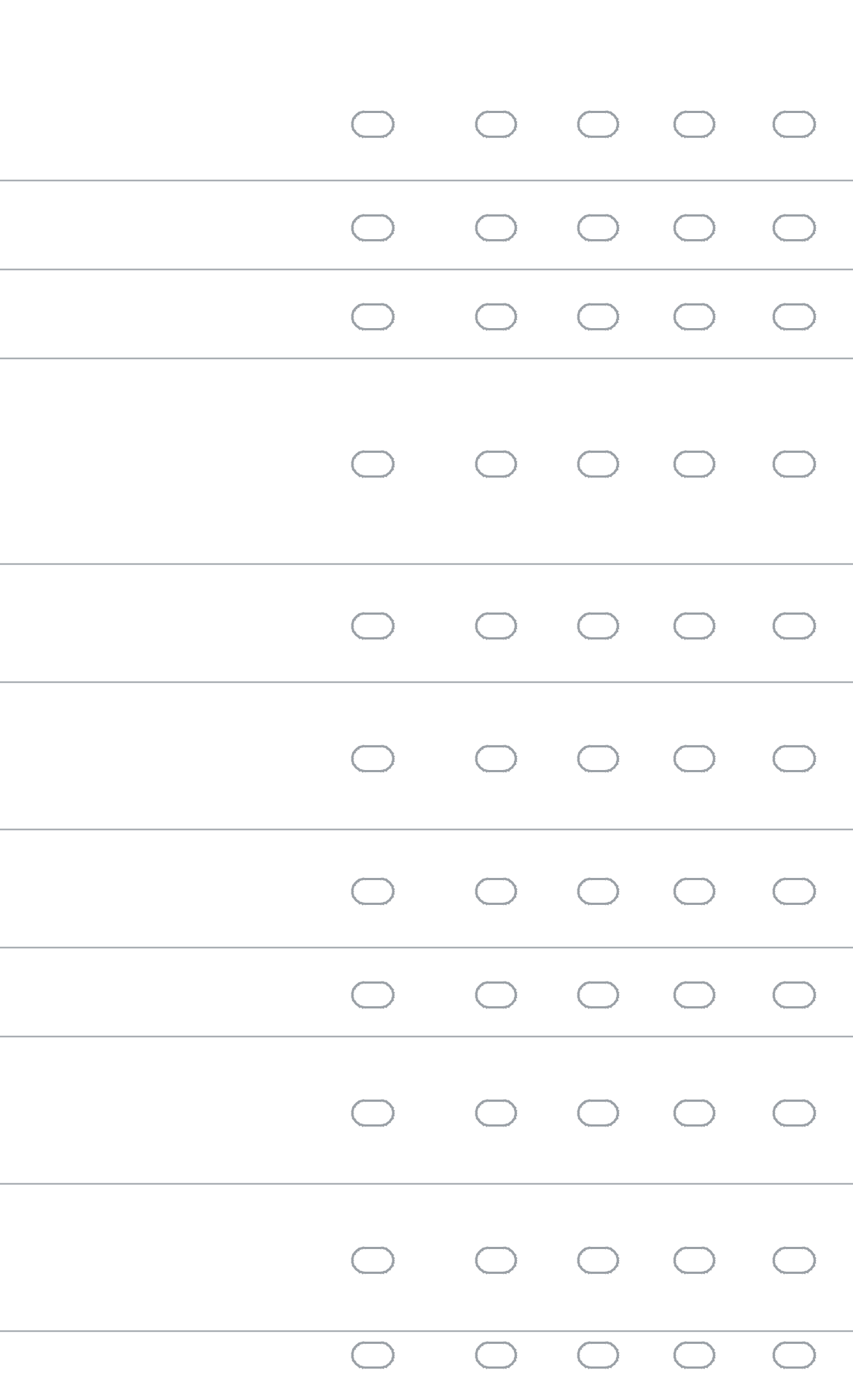
***

| Strongly | Disagree Neutral | Agree | Strongly |
| --- | --- | --- | --- |
| disagree |  |  | agree |
|  |  |  |  |
|  |  |  |  |

I **am** i**nvo**l**ved w**i**th recru**i**tment**

**of pat**i**ents to research stud**i**es**

**at my centre**

**My tra**i**n**i**ng env**i**ronment**

**promotes research act**i**v**i**ty**

I **have support for research**

**pursu**i**ts at my centre**

**Rather than comp**l**ete a**

**research pro**j**ect**,, I **wou**l**d**

**prefer for comp**l**ete another**

**educat**i**ona**l **act**i**v**i**ty (**i**e**.

**arthrop**l**asty advance course**,,

**trauma surg**i**ca**l **course**,, **etc** ..**)**

I **on**l**y do research because** i**t**

i**s a mandatory part of my**

**res**i**dency program**

I **wou**l**d prefer to deve**l**op my**

**own research quest**i**on than**

**work on a pro**j**ect that** i**s**

**a**l**ready ongo**i**ng**

**My current research pro**j**ect**

**was an** i**dea that my**

**superv**i**sor proposed**

**Res**i**dent research** i**s a pos**i**t**i**ve**

**exper**i**ence**

I**nvo**l**vement** i**n res**i**dent**

**research made me more** li**ke**l**y**

**to ass**i**st** i**n future research**

**pro**j**ects**

I**nvo**l**vement** i**n res**i**dent**

**research made me more** li**ke**l**y**

**to** i**n**i**t**i**ate future research**

**pro**j**ects**

I**nvo**l**vement** i**n res**i**dent**

**research has** i**ncreased my**


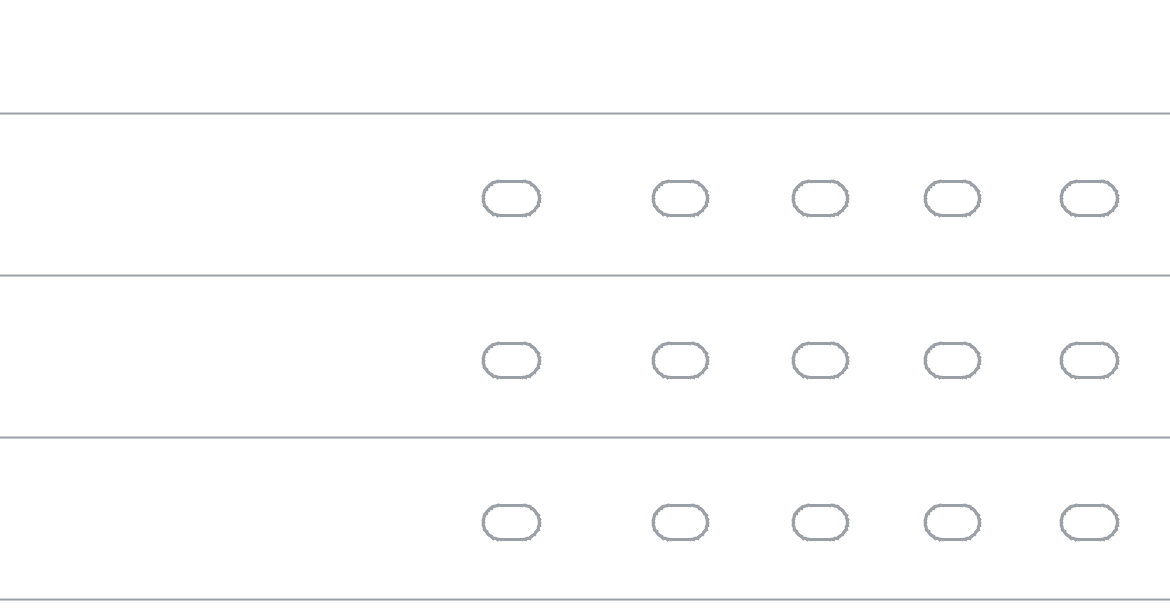


**des**i**re to pursue research as a**

**staff** l**eve**l **phys**i**c**i**an**

**A research pro**j**ect shou**l**d be**

**mandatory** i**n Orthoped**i**c**

**res**i**dency**

**Research act**i**v**i**t**i**es are**

i**mportant to gu**i**de our**

**pract**i**ce**

**Understand**i**ng the** li**terature** i**s**

**an** i**mportant part of my future**

**pract**i**ce**

**
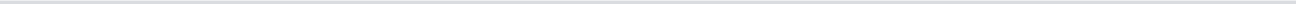
**
